# Impact of germline polymorphisms in genes regulating glucose uptake on positron emission tomography findings and outcome in diffuse large B-cell lymphoma: results from the PETAL trial

**DOI:** 10.1007/s00432-021-03796-z

**Published:** 2021-10-27

**Authors:** Martina Broecker-Preuss, Nina Becher-Boveleth, Stefan P. Müller, Andreas Hüttmann, Christine Hanoun, Hong Grafe, Julia Richter, Wolfram Klapper, Jan Rekowski, Andreas Bockisch, Ulrich Dührsen

**Affiliations:** 1 Klinik für Nuklearmedizin, Universitätsklinikum Essen, Hufelandstraße 55, 45147 Essen, Germany; 2 Zentrallabor, Universitätsklinikum Essen, Essen, Germany; 3 Klinik für Hämatologie, Universitätsklinikum Essen, Essen, Germany; 4 Sektion für Hämatopathologie, Universitätsklinikum Schleswig-Holstein, Campus Kiel, Kiel, Germany; 5 Institut für Medizinische Informatik, Biometrie und Epidemiologie, Universität Duisburg-Essen, Essen, Germany

**Keywords:** Diffuse large B-cell lymphoma, Glucose metabolism, Outcome, Positron emission tomography, Single nucleotide polymorphism, Standardized uptake value, Total metabolic tumour volume

## Abstract

**Background:**

[^18^F]Fluoro-deoxyglucose (FDG) positron emission tomography/computed tomography (PET/CT) is the standard imaging procedure in diffuse large B-cell lymphoma (DLBCL). Disease presentation, FDG-PET/CT performance, and outcome may be influenced by germline single nucleotide polymorphisms (SNP) in genes regulating glucose uptake.

**Methods:**

Clinical variables, FDG-PET findings, and outcome were analysed in relation to SNPs in 342 DLBCL patients participating in the ‘Positron Emission Tomography-Guided Therapy of Aggressive Non-Hodgkin Lymphomas’ (PETAL) trial. Genes analysed included SLC2A1 (SNPs rs1385129, referred to as HaeIII; rs710218, HpyCH4V; rs841853, XbaI), VEGFA (rs3025039), HIF1A (rs11549465, P582S; rs11549467, A588T), and APEX1 (rs1130409, D148E). Statistical significance was assumed at *p* ≤ 0.05.

**Results:**

The SLC2A1 HaeIII and HpyCH4V SNPs were tightly linked and statistically significantly associated with baseline maximum standardized uptake value (SUV_max_) and Ann Arbor stage, with slightly lower SUV_max_ (HaeIII, median 18.9, interquartile range [IQR] 11.5–26.6, versus 21.6, IQR 14.4–29.7; *p* = 0.019) and more frequent stage IV disease (HaeIII, 44.5% versus 30.8%; *p* = 0.011) in minor allele carriers. As previously reported for lung cancer, the association was dependent upon the coexistent APEX1 D148E genotype. The HIF1A A588T SNP was associated with total metabolic tumour volume (TMTV) and time-to-progression, with significantly lower TMTV (median 16 cm^3^, IQR 7–210, versus 146 cm^3^, IQR 34–510; *p* = 0.034) and longer time-to-progression in minor allele carriers (log-rank *p* = 0.094). Time-to-progression was also associated with the SLC2A1 XbaI and APEX1 D148E SNPs, with shorter time-to-progression in homozygous and heterozygous SLC2A1 XbaI (HR 1.456; CI 0.930–2.280; *p* = 0.099) and homozygous APEX1 D148E minor allele carriers (HR 1.6; CI 1.005–2.545; *p* = 0.046). In multivariable analyses including SNPs, International Prognostic Index factors, sex, and B symptoms, HIF1A A588T, SLC2A1 XbaI, and APEX1 D148E retained statistical significance for time-to-progression, and SLC2A1 XbaI was also significantly associated with overall survival.

**Conclusions:**

Common SNPs in genes regulating glucose uptake may impact SUV_max_, tumour distribution, tumour volume, and outcome in DLBCL. The effects on SUV_max_ are of low magnitude and appear clinically negligible. The results are consistent with findings in other types of cancer. They need to be confirmed in an independent DLBCL population of sufficient size.

**Trial registration:**

Trial registration: ClinicalTrials.gov NCT00554164; EudraCT 2006-001641-33. Registration date November 5, 2007, https://www.clinicaltrials.gov/ct2/show/NCT00554164

**Supplementary Information:**

The online version contains supplementary material available at 10.1007/s00432-021-03796-z.

## Background

Diffuse large B-cell lymphoma (DLBCL) is the most frequent cancer of the immune system (Morton et al. 2006). [^18^F]Fluoro-deoxyglucose (FDG) positron emission tomography/computed tomography (PET/CT) is the standard imaging procedure to define tumour stage and treatment response (Cheson et al. 2014). Because stage and response may impact therapy (Poeschel et al. 2020; Persky et al. 2020), reliable FDG-PET/CT performance is of utmost importance.

The uptake of glucose and FDG is controlled by several genes. One of the most important ones is solute carrier family 2 member 1 (SLC2A1, also known as GLUT1, facilitated glucose transporter type 1) encoding the major channel for glucose entry into the cell (Ancey et al. 2018). The expression of SLC2A1 is stimulated by the transcription factor hypoxia-inducible factor 1α (gene HIF1A), the major regulator of hypoxic responses (Lee et al. 2019). Another HIF1α target gene is VEGFA whose protein product, vascular endothelial growth factor A, induces blood vessel formation, thus increasing glucose supply (Apte et al. 2019). The activity of HIF1α is enhanced by apurinic/apyrimidinic endonuclease 1 (APEX1), a multifunctional protein involved in DNA repair and transcription factor activation (Shah et al. 2017). Single nucleotide polymorphisms (SNP) in these genes have been studied extensively with regard to risk, presentation, and outcome of cancer (Amann et al. 2011; Feng et al. 2017; Heist et al. 2008; Kim et al. 2012; Tanimoto et al. 2003; Knechtel et al. 2010; Qin et al. 2012; Hill et al. 2006; Matakidou et al . 2007; Smith et al. 2008; Cao et al. 2011). In some studies, their impact on FDG-PET performance was also evaluated (Wolf et al. 2004; Grabellus et al. 2010; Kim et al. 2010; Bravatà et al. 2013).

We investigated the impact of SNPs in the named four genes on DLBCL presentation, FDG-PET findings, and outcome in the setting of the ‘Positron Emission Tomography-Guided Therapy of Aggressive Non-Hodgkin Lymphomas’ (PETAL) trial (Dührsen et al. 2018). The trial set out – and failed – to improve treatment by adapting it to the response to the first two cycles of therapy. Because interim PET-driven treatment changes had no impact on outcome, all treatment arms were combined in the present analysis.

## Methods

### Study design

The PETAL trial (ClinicalTrials.gov NCT00554164; EudraCT 2006-001641-33) was a multicentre study for newly diagnosed aggressive non-Hodgkin lymphomas (Dührsen et al. 2018). Diagnoses were confirmed by reference pathological review including molecular analyses (Richter et al. 2019). The study was approved by the Federal Institute for Drugs and Medical Devices and the ethics committees of the participating sites. All patients gave written informed consent including permission of data use for post hoc scientific analyses.

Patients were treated with rituximab, cyclophosphamide, doxorubicin, vincristine, and prednisone (R-CHOP). Interim PET was performed a median of 20 days after cycle 2. Patients with favourable response received four more cycles of R-CHOP or the same treatment plus two extra doses of rituximab. Patients with unfavourable response were randomly assigned to receive six additional cycles of R-CHOP or six blocks of a more intensive protocol originally developed for Burkitt’s lymphoma (Dührsen et al. 2018).

### FDG-PET/CT imaging and evaluation

The imaging conditions have been described previously (Dührsen et al. 2018). The treatment response was determined by dividing the maximum standardized uptake value (SUV_max_) of the hottest residual lesion on the interim scan by the SUV_max_ of the hottest lesion on the baseline scan. Favourable scans were defined by complete disappearance of all non-physiological FDG activity or SUV_max_ reduction by > 66% (Rekowski et al. 2021). During the trial, the treatment response was assessed by local investigators. A post hoc central review yielded a concordance of 97.7% with the original assessment (Dührsen et al. 2018). Total metabolic tumour volume (TMTV) was determined centrally on archived PET/CT scans, using the 41% SUV_max_ method (Schmitz et al. 2020). End-of-treatment responses were defined by CT criteria (Cheson et al. 2014; Dührsen et al. 2018).

### Single nucleotide polymorphisms

The analysis was restricted to glucose uptake-regulating genes whose SNPs had previously been shown to be associated with FDG-PET findings (Wolf et al. 2004; Grabellus et al. 2010; Kim et al. 2010) or tumour mass (Tanimoto et al. 2003; Knechtel et al. 2010; Qin et al. 2012). Germline SNPs in genomic DNA from peripheral blood mononuclear cells were detected by allele-specific polymerase chain reaction (PCR) using Assay-on-demand TaqMan SNP assays that contained primers and allele-specific VIC- and FAM-labelled probes and genotyping master mix (Thermo Fisher Scientific, Waltham, MA, USA). Cycling conditions were 60 °C for 30 s and an initial denaturation step of 95 °C for 10 min followed by 40 cycles of 95 °C for 10 s and 60 °C for 30 s using StepOne Plus real-time PCR instrumentation (Thermo Fisher Scientific). Allele typing was performed using the StepOne Software. In every PCR plate, negative controls as well as internal positive controls were included for homozygous and heterozygous sample types.

### HIF1A expression

Expression of the HIF1α protein was studied by immunohistochemistry using the antibody H1alpha67 (Novus Biologicals, Abingdon, United Kingdom; 1:50, pH 9), with normoxic and hypoxic BL-70 cells serving as negative and positive controls, respectively. To induce hypoxia, BL-70 cells were grown in the presence of 100 µM CoCl_2_ for 20 h at 37 °C in 5% CO_2_ in air, followed by preparation of cell pellets in formalin-fixed paraffin-embedded blocks.

### Statistical analysis

All analyses were exploratory, applying a two-sided alpha of 0.05. Numerical variables and frequencies were compared using the Kruskal–Wallis and chi^2^ tests, respectively, and time-to-event end-points were analysed using the Kaplan–Meier estimator, the log-rank test, and, when adjusting for covariates, Cox proportional hazards regression. Results were not corrected for multiple testing. All analyses were carried out using IBM SPSS Statistics, version 26.0, Armonk, NY, USA.

## Results

### Patient characteristics

Of 862 patients treated in the PETAL trial, 609 had DLBCL (Dührsen et al. 2018). Peripheral blood for genotyping was collected from 342 patients (Table 1). Favourable and unfavourable interim PET responses were recorded in 308 (90.1%) and 34 patients (9.9%), respectively.

**Table 1:** Patient characteristics

	Number	Percent		Number	Percent
Total number of patients	342	100%	B symptoms	96	28.1%
Age—median (range), years	60 (18–79)	n.a	Weight loss > 10% in 6 months	49	14.3%
Male sex	181	52.9%	Drenching night sweats	63	18.4%
Female sex	161	47.1%	Fever	22	6.4%
**International Prognostic Index factors**			**Treatment allocation**		
Age > 60 years	167	48.8%	6xCHOP^a^	1	0.3%
ECOG performance status ≥ 2	35	10.2%	6xR-CHOP	142	41.5%
Ann Arbor stage III or IV	192	56.2%	6xR-CHOP + 2xR	170	49.7%
Extranodal sites > 1	105	30.7%	8xR-CHOP	15	4.4%
Lactate dehydrogenase > ULN	171	50.0%	2xR-CHOP + 6 × Burkitt protocol	14	4.1%
**International Prognostic Index**			**End-of-treatment response**		
Low risk	142	41.5%	Complete remission	136	39.8%
Low-intermediate risk	79	23.1%	Partial remission	178	52.0%
High-intermediate risk	77	22.5%	Stable disease	24	7.0%
High risk	44	12.9%	Progressive disease	1	0.3%

*CHOP* cyclophosphamide, vincristine, doxorubicin, prednisone, *ECOG* Eastern Cooperative Oncology Group, *R* rituximab, *ULN* upper limit of normal, *n.a.* not applicable

^a^CD20-negative lymphoma

SUV_max_ and TMTV were determined in 341 and 298 patients, respectively. Median baseline SUV_max_ was 20.5 (interquartile range [IQR], 13.3–28.8), median SUV_max_ reduction at interim scanning was 83.1% (IQR, 73.1–88.5), and median baseline TMTV was 140 cm^3^ (IQR, 33–494). TMTV was below or above the previously defined prognostic threshold of 328 cm^3^ (Schmitz et al. 2020) in 196 (65.8%) and 102 patients (34.2%), respectively.

Reference pathological review was performed in 337 patients (98.5%). Cell-of-origin-based subtyping using the Hans classifier (Hans et al. 2004) was available for 162 lymphomas, showing germinal centre B-cell (GCB) and non-GCB lymphomas in 80 (49.4%) and 82 cases (50.6%), respectively. The double-hit status (simultaneous translocations of *MYC* and *BCL2* or *BCL6*) was determined in 146 lymphomas, with a positive result in 11 cases (7.5%).

### SNP characteristics

Genotyping included seven commonly studied SNPs in SLC2A1 (rs1385129, subsequently referred to as HaeIII; rs710218, HpyCH4V; rs841853, XbaI), VEGFA (rs3025039), HIF1A (rs11549465, P582S; rs11549467, A588T), and APEX1 (rs1130409, D148E). Genotypes and allele frequencies are displayed in the Supplementary Information, Supplementary Table 1. Deviations from the Hardy–Weinberg equilibrium were not observed. The SLC2A1 HaeIII and HpyCH4V SNPs were tightly linked, with 338 of 342 patients (98.8%) showing identical allelic distributions.

### SNPs and baseline clinical features

Sex, age, performance status, extranodal disease, serum lactate dehydrogenase, B symptoms (as dichotomized in Table 1), histological bone marrow involvement, International Prognostic Index, cell-of-origin and double-hit status failed to show an association with any of the SNPs tested.

### SNPs and FDG-PET/CT findings

The SLC2A1 HaeIII and HpyCH4V genotypes were statistically significantly associated with baseline SUV_max_, with lower values in minor allele carriers than in homozygous major allele carriers (HaeIII, median 18.9 versus 21.6, *p* = 0.019; HpyCH4V, 19.0 versus 21.4, *p* = 0.030; Table 2). The association of the SLC2A1 HpyCH4V genotype with SUV_max_ has previously been shown to be dependent upon the APEX1 D148E genotype (Kim et al. 2010). In line with this observation, the association of SLC2A1 HaeIII and HpyCH4V genotypes with SUV_max_ was restricted to homozygous APEX1 major allele carriers (Supplementary Table 2). Irrespective of the APEX1 genotype, none of the other SNPs including SLC2A1 XbaI showed an association with baseline SUV_max_ (Supplementary Tables 2 and 3).

**Table 2:** Association between SLC2A1 HaeIII and HpyCH4V genotypes and baseline maximum standardized uptake value

Polymorphism (rs number)	Genotype	No. pts.	Median SUV_max_ (IQR)	p	Dominant model	No. pts.	Median SUV_max_ (IQR)	p	Recessive model	No. pts.	Median SUV_max_ (IQR)	p
SLC2A1 HaeIII (rs1385129)	CC CT TT	214 108 19	21.6 (14.4–29.7) 18.6 (11.5–25.5) 22.4 (11.7–30.1)	0.041	CC CT + TT	214 127	21.6 (14.4–29.7) 18.9 (11.5–26.6)	0.019	CC + CT TT	322 19	20.5 (13.3–28.6) 22.4 (11.7–30.1)	0.878
SLC2A1 HpyCH4V (rs710218)	AA AT TT	210 111 20	21.4 (14.2–29.9) 18.7 (11.5–25.5) 22.8 (12.6–29.9)	0.055	AA AT + TT	210 131	21.4 (14.2–29.9) 19.0 (11.5–26.6)	0.030	AA + AT TT	321 20	20.4 (13.3–28.6) 22.8 (12.6–29.9)	0.767

*No. pts.* number of patients, *SUV*_*max*_ maximum standardized uptake value, *IQR* interquartile range (25th–75th percentile), *p* Kruskal–Wallis test

The SLC2A1 HaeIII and HpyCH4V genotypes were also significantly associated with Ann Arbor stage (chi^2^ test, *p* = 0.006 and *p* = 0.005, respectively), driven by a higher number of stage IV patients among minor allele carriers than among homozygous major allele carriers (HaeIII, 44.5% versus 30.8%, *p* = 0.011; HpyCH4V, 44.7% versus 30.5%, *p* = 0.008; Table 3). The association was restricted to homozygous or heterozygous APEX1 major allele carriers (Supplementary Table 4). None of the other SNPs showed an association with Ann Arbor stage (Supplementary Tables 4 and 5).

**Table 3:** Association between SLC2A1 HaeIII and HpyCH4V genotypes and Ann Arbor stage IV disease

Polymorphism (rs number)	Genotype	No. pts.	Ann Arbor Stage I-III vs. IV	p	Dominant model	No. pts.	Ann Arbor Stage I-III vs. IV	p	Recessive model	No. pts.	Ann Arbor Stage I-III vs. IV	p
SLC2A1 HaeIII (rs1385129)	CC CT TT	214 109 19	148 vs.66 58 vs. 51 13 vs. 6	0.017	CC CT + TT	214 128	148 vs. 66 71 vs. 57	0.011	CC + CT TT	323 19	206 vs. 117 13 vs. 6	0.682
SLC2A1 HpyCH4V (rs710218)	AA AT TT	210 112 20	146 vs. 64 60 vs. 52 13 vs. 7	0.018	AA AT + TT	210 132	146 vs. 64 73 vs. 59	0.008	AA + AT TT	322 20	206 vs. 116 13 vs. 7	0.926

*No. pts.* number of patients, *p* chi^2^ test

**Fig. 1:**
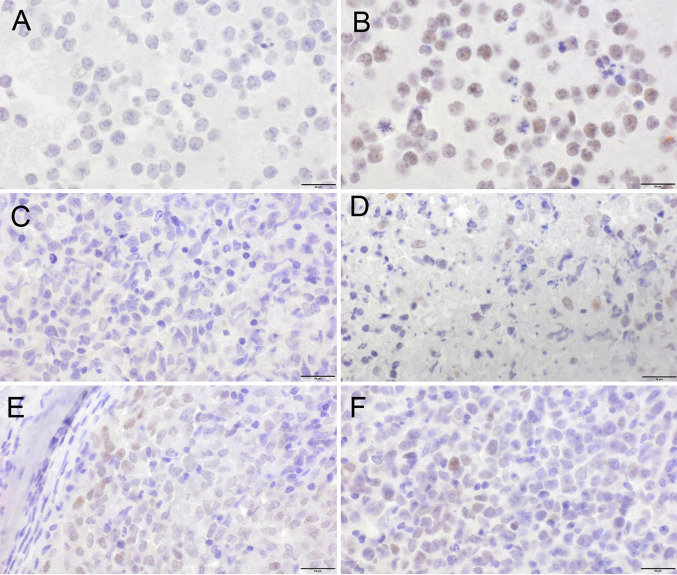
HIF1α protein expression by immunohistochemistry using the H1alpha67 antibody. **A** No expression in normoxic, and **B** strong expression (brown) in hypoxic BL-70 controls. **C** No expression in the majority of tumour cells in a heterozygous HIF1A A588T minor allele carrier, with (**D**), some positive cells in an area close to necrosis. **E** < 1% scattered positive cells, and **F** < 10% positive cells in homozygous HIF1A A588T major allele carriers. The bar is equal to 20 µm

Baseline TMTV was associated with the HIF1A A588T SNP, with significantly lower volumes in minor allele carriers. Because data on the SNP’s functional consequences are scant, we studied HIF1α expression by immunohistochemistry in tumour samples from major and minor allele carriers. One of 4 minor allele carriers and 3 of 16 homozygous major allele controls matched for TMTV (median, 17 cm^3^ vs. 18 cm^3^; range, 6–89 vs. 4–147), cell-of-origin, and double-hit status showed weak staining for HIF1α (*p* = 1, Fisher’s exact test; Fig. 1). A statistically significant association was also found between TMTV and the HIF1A P582S genotype, but this finding was not further pursued, as it was based on only two patients (Table 4). None of the other SNPs was correlated with TMTV (Supplementary Table 6).

**Table 4:** Association between HIF1A genotypes and baseline total metabolic tumor volume

Polymorphism (rs number)	Genotype	No. pts.	Median TMTV in cm^3^ (IQR)	p	Dominant model	No. pts.	Median TMTV in cm^3^ (IQR)	p	Recessive model	No. pts.	Median TMTV in cm^3^ (IQR)	p
HIF1A P582S (rs11549465)	CC CT TT	249 47 2	145 (32–474) 107 (37–565) 325 (n.a.)	0.934	CC CT + TT	249 49	145 (32–474) 107 (34–582)	0.792	CC + CT TT	296 2	140 (33–490) 325 (n.a.)	0.042
HIF1A A588T (rs11549467)	GG GA AA	290 8 0	146 (34–510) 16 (7–210) n.a	0.034	GG GA + AA	290 8	146 (34–510) 16 (7–210)	0.034	GG + GA AA	298 0	140 (33–494) n.a	n.a

*No. pts.* number of patients, *TMTV* total metabolic tumor volume, *IQR* interquartile range (25th–75th percentile), *p* Kruskal–Wallis test, *n.a.* not applicable

SUV_max_ reduction at interim scanning and interim PET response failed to show an association with SNPs (Supplementary Tables 7 and 8).

### SNPs and outcome

**Fig. 2:**
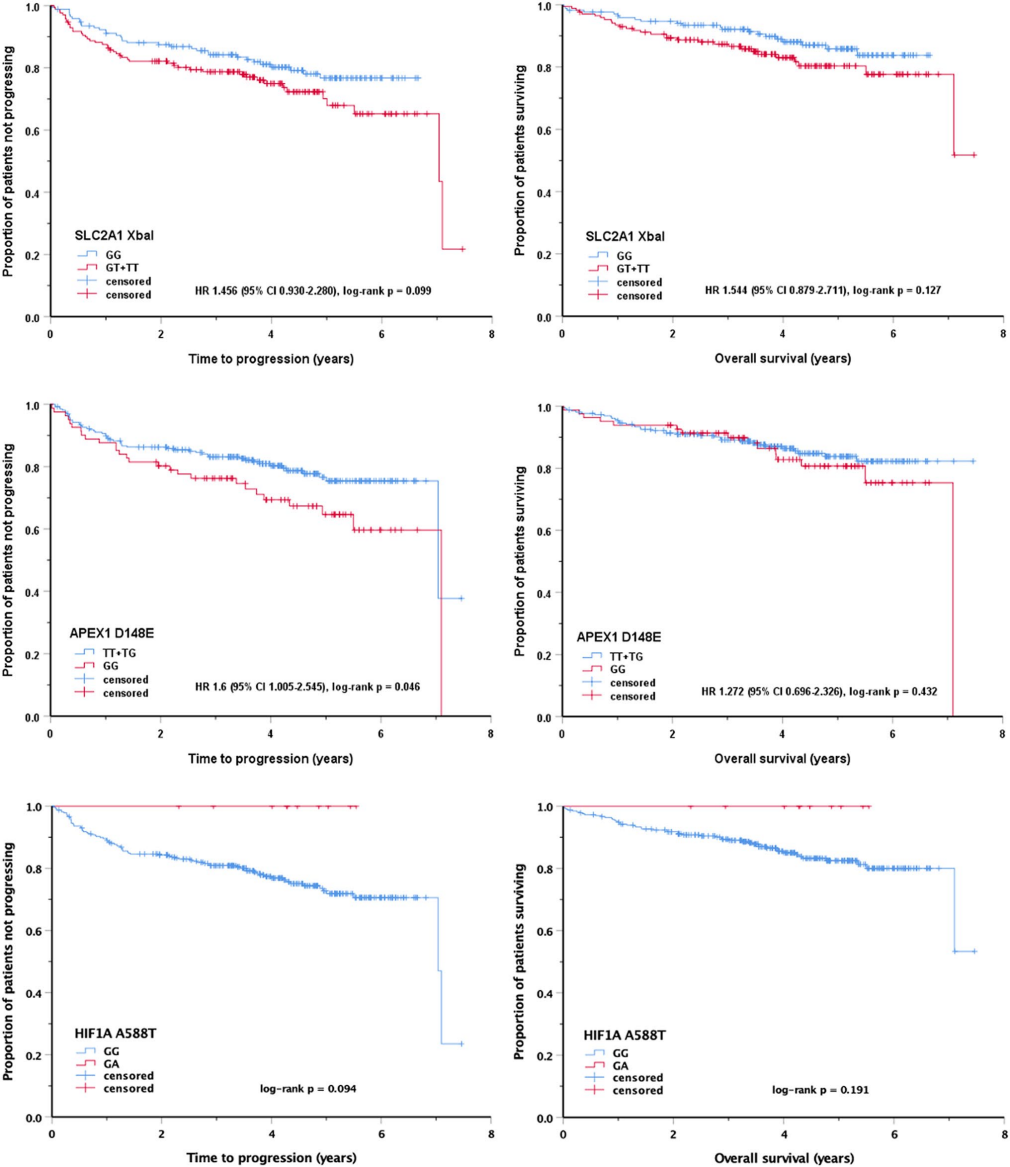
Impact of single nucleotide polymorphisms on time-to-progression and overall survival (HR, hazard ratio; CI, confidence interval)

Outcome measures included end-of-treatment remission status, time-to-progression, and overall survival. There was no correlation between SNPs and remission status (Table 1). With a median follow-up of 52 months, a trend (*p* < 0.1) for reduced time-to-progression was observed in homozygous or heterozygous minor allele carriers of the SLC2A1 XbaI SNP and in homozygous minor allele carriers of the APEX1 D148E SNP (Fig. 2). Time-to-progression was prolonged in minor allele carriers of the HIF1A A588T SNP. Overall survival showed a weak correlation with SLC2A1 XbaI and HIF1A A588T, which was more pronounced in homozygous SLC2A1 XbaI minor allele carriers (*p* = 0.082; data not shown).

To investigate the SNPs’ impact in relation to other variables, they were entered into multivariable Cox regression analyses with stepwise backward elimination of factors failing statistical significance. The first step included SNPs, sex, age, performance status, Ann Arbor stage, extranodal disease, serum lactate dehydrogenase, and B symptoms as dichotomized in Table 1. The final models were dominated by clinical variables, but SLC2A1 XbaI, APEX1 D148E, and HIF1A A588T were retained in the time-to-progression model and SLC2A1 XbaI was retained in the overall survival model (Table 5). Similar results were obtained when the model included the International Prognostic Index risk groups (Table 1) instead of the five factors defining the index (data not shown).

**Table 5:** Cox model for time-to-progression and overall survival

	Hazard ratio	95% confidence interval	*P*
**Time-to-progression**			
Age > 60 years	1.590	1.014–2.494	0.042
Ann Arbor stage III or IV	2.653	1.512–4.654	< 0.001
Lactate dehydrogenase > ULN	1.864	1.125–3.090	0.013
B symptoms	1.627	1.023–2.589	0.043
SLC2A1 XbaI, GT/TT genotypes	1.698	1.081–2.667	0.021
APEX1 D148E, GG genotype	1.651	1.037–2.629	0.041
HIF1A A588T, GA genotype	0.000	–	0.042
**Overall survival**			
Age > 60 years	2.677	1.477–4.854	0.001
Ann Arbor stage III or IV	2.002	1.006–3.983	0.039
Lactate dehydrogenase > ULN	2.288	1.182–4.427	0.010
B symptoms	2.282	1.282–4.063	0.005
SLC2A1 XbaI, GT/TT genotypes	1.902	1.078–3.354	0.025

*ULN* upper limit of normal, *p* likelihood ratio test

When TMTV dichotomized at 328 cm^3^ (Schmitz et al. 2020) was added to a model restricted to patients with available TMTV data, HIF1A A588T lost its significance for time-to-progression (*p* = 0.118), while SLC2A1 XbaI and APEX1 D148E were retained at borderline significance (*p* = 0.054). SLC2A1 XbaI was also retained in the overall survival model (*p* = 0.014; Supplementary Table 9).

## Discussion

Key findings of our study include the association of the SLC2A1 HaeIII and HpyCH4V SNPs with baseline SUV_max_ and Ann Arbor stage, the association of the HIF1A A588T SNP with TMTV, and the association of the HIF1A A588T, SLC2A1 XbaI and APEX1 D148E SNPs with long-term outcome.

The SLC2A1 HaeIII and HpyCH4V SNPs were tightly linked, yielding almost identical results. The HpyCH4V SNP is located in the SLC2A1 promotor region in proximity to a putative hypoxic response element which may affect HIF1α binding and SLC2A1 expression (Feng et al. 2017). The minor allele was found to be associated with increased expression in colorectal cancer (Feng et al. 2017) and decreased expression in hepatocellular carcinoma (Amann et al. 2011). In breast cancer (Grabellus et al. 2010; Bravatà et al. 2013) and non-small cell lung cancer (Kim et al. 2010), baseline SUV_max_ was not affected by the SNPs, but when the analysis was restricted to lung cancer patients homozygous for the APEX1 D148E major allele, homozygosity for the SLC2A1 HpyCH4V minor allele was significantly associated with increased SUV_max_ (Kim et al. 2010). The APEX1 genotype dependency of the SLC2A1 SNPs’ association with SUV_max_ was confirmed in our study. However, the variant alleles were associated with decreased rather than increased SUV_max_, both in homozygous and heterozygous patients. As pointed out above, the direction in which the variant allele shifts SLC2A1 expression varies from cancer to cancer (Amann et al. 2011; Feng et al. 2017). This may also hold true for the SLC2A1/APEX1 interaction whose nature has yet to be elucidated. In contrast to HaeIII and HpyCH4V, the SLC2A1 XbaI SNP was not correlated with SUV_max_. This finding is at variance with a breast cancer study, where homozygosity for the major allele was associated with increased SUV_max_ (Grabellus et al. 2010).

The SLC2A1 HaeIII and HpyCH4V SNPs were also associated with Ann Arbor stage, a well-established measure of lymphoma distribution (Cheson et al. 2014). Stage IV was overrepresented in HaeIII and HpyCH4V minor allele carriers, provided they also carried the APEX1 major allele. This unexpected association would find an explanation if disseminated disease were more readily detectable in HaeIII or HpyCH4V minor allele carriers. Two observations argue against this assumption. First, SUV_max_ was lower in minor allele carriers than in homozygous major allele carriers, making improved detectability of low intensity lesions unlikely. Second, in contrast to the association with SUV_max_, the association with stage IV disease was seen in both homozygous and heterozygous APEX1 major allele carriers, suggesting distinct biological principles.

Minor allele carriers of the HIF1A A588T SNP had significantly reduced TMTV, a measure of tumour mass. Irrespective of genotype, HIF1α protein expression was undetectable or weak by immunohistochemistry. Since the amino acid substitution has been reported to increase the protein’s transactivation capacity, enhanced function does not require increased expression (Tanimoto et al. 2003). HIF1α-mediated effects on tumour mass have been demonstrated previously (Schwab et al. 2012). Similar to our observation, renal cell carcinoma patients with variant HIF1A alleles including A588T were shown to present in low tumour stages (Qin et al. 2012). By contrast, in head-and-neck and colorectal cancer, tumour size was found to be increased (Tanimoto et al. 2003; Knechtel et al. 2010). The direction of the effect of a variant HIF1A allele on tumour mass appears to be cancer type-specific.

To assess the SNPs’ impact on long-term outcome, we chose two non-overlapping endpoints. Time-to-progression best captures disease-related features, such as disease course and response to therapy. Overall survival is dominated by patient features, such as age, comorbidities, and disease and treatment tolerance (Schmitz et al. 2020). The HIF1A A588T minor allele had a pronounced effect on both endpoints, but this observation was based on only ten patients. Importantly, the SNP’s impact on time-to-progression lost its significance, when TMTV was included in the prognostic model, suggesting that the minor allele’s beneficial effect on outcome was mediated by low tumour mass. In line with our observation, survival was also prolonged in renal cell carcinoma patients carrying a variant allele (Qin et al. 2012). Irrespective of SNPs, detectable HIF1α expression has been reported to portend a favourable prognosis in DLBCL (Evens et al. 2010). In the small set studied here, none of 4 patients with and 2 of 16 patients without detectable HIF1α expression progressed (time-to-progression, log-rank *p* = 0.517; data not shown).

Time-to-progression was also affected by SLC2A1 XbaI and APEX1 D148E. With regard to the former, a detrimental effect of the minor allele was demonstrable in both homozygous and heterozygous carriers, while with the latter, this was restricted to homozygous patients. In contrast to all other SNPs, XbaI not only influenced time-to-progression, but also overall survival. To our knowledge, the biological consequences of the XbaI SNP are unknown, and SNP-related outcome data are not available for other types of cancer. As for APEX1 D148E, the amino acid substitution has been associated with reduced DNA repair and increased genotoxic stress (Lirussi et al. 2016). This is associated with a predisposition for solid tumours (Smith et al. 2008; Cao et al. 2011), but not non-Hodgkin lymphoma (Hill et al. 2006). Functional data on HIF1α regulation by the variant APEX1 protein is lacking. In lung cancer patients homozygous for the APEX1 D148E minor allele, overall survival was reported to be prolonged rather than reduced, again demonstrating differences between cancer types (Matakidou et al 2007).

The VEGFA SNP failed to show a correlation with any of the variables tested. The minor allele is associated with reduced VEGF expression (Wolf et al. 2004), which may explain why its effect on some types of cancer is favourable (Heist et al. 2008; Wolf et al. 2004). Similar to our findings, no impact on outcome was observed in a previous DLBCL study (Kim et al. 2012).

Strengths of our study include its large sample size, prospective nature, and rigorously controlled conditions of PET performance and treatment delivery. Its major limitation is the multitude of SNPs and variables tested. Most of the associations would have failed to reach statistical significance, had they been corrected for multiple testing. Thus, our results are hypothesis-raising rather than definitive. Most of them, however, are consistent with previous findings. SNPs in SLC2A1 influence SUV_max_. Though statistically significant, the extent of SNP-related variability appears clinically negligible in DLBCL, not exceeding the expected test–retest variation for SUV measurements (Kurland et al. 2019). SNPs in HIF1A are related to tumour mass, a major determinant of disease outcome (Schmitz et al. 2020). APEX1 D148E is also related to outcome, possibly mediated by disturbed DNA repair. Finally, the direction of the SNP effects on the investigated variables appears to be cancer type-specific. The reason for this observation remains to be elucidated.

## Conclusions

Common SNPs in genes regulating glucose uptake may impact SUV_max_, tumour distribution, tumour volume, and outcome in DLBCL. The results are consistent with findings in other types of cancer. They need to be confirmed in an independent DLBCL population of sufficient size.

## Supplementary Information

Below is the link to the electronic supplementary material.

Supplementary file1 (DOCX 57 kb)

## Data Availability

The datasets used and analysed during this study are available from the corresponding author on reasonable request.

## Abbreviations

APEX1: Apurinic/apyrimidinic endonuclease 1
CT: Computed tomography
DLBCL: Diffuse large B-cell lymphoma
FDG: [^18^F]fluoro-deoxyglucose
GCB: Germinal centre B-cell
GLUT1: Facilitated glucose transporter type 1
HIF1A: Hypoxia-inducible factor 1A (gene)
HIF1α: Hypoxia-inducible factor 1α (protein)
IQR: Interquartile range
PCR: Polymerase chain reaction
PET: Positron emission tomography
PETAL: Positron Emission Tomography-Guided Therapy of Aggressive Non-Hodgkin Lymphomas
R-CHOP: Rituximab, cyclophosphamide, doxorubicin, vincristine, prednisone
SLC2A1: Solute carrier family 2 member 1
SNP: Single nucleotide polymorphism
SUV: Standardized uptake value
SUV_max_: Maximum standardized uptake value
TMTV: Total metabolic tumour volume
VEGFA: Vascular endothelial growth factor A
