# Supplementary material for: Impact of germline polymorphisms in genes regulating glucose uptake on positron emission tomography findings and outcome in diffuse large B-cell lymphoma: results from the PETAL trial
Source: J Cancer Res Clin Oncol. 2021 Oct 27;148(10):2611–21. doi: 10.1007/s00432-021-03796-z (PMC9470686; doi:10.1007/s00432-021-03796-z)
Supplement: Supplementary file 1 — Supplementary file1 (DOCX 57 kb) [file 432_2021_3796_MOESM1_ESM.docx]

# Supplementary Information

**Impact of germline polymorphisms in genes regulating glucose uptake on positron emission tomography findings and outcome in diffuse large B-cell lymphoma: results from the PETAL trial**

Martina Broecker-Preuss, Nina Becher-Boveleth, Stefan P. Müller, Andreas Hüttmann, ChristineHanoun, Hong Grafe, Julia Richter, Wolfram Klapper, Jan Rekowski, Andreas Bockisch & Ulrich Dührsen

Supplementary Tables 1 - 9

1. Genotypes and allele frequencies

2. Association between genotypes and baseline maximum standardized uptake value

3. APEX1 D148E genotype-dependent association between SLC2A1 genotypes and baseline maximum standardized uptake value

4. Association between genotypes and Ann Arbor stage IV disease

5. APEX1 D148E genotype-dependent association between SLC2A1 genotypes and Ann Arbor stage IV disease

6. Association between genotypes and baseline total metabolic tumor volume

7. Association between genotypes and relative standardized uptake value reduction at interim positron emission tomography

8. Association between genotypes and interim positron emission tomography response

9. Cox model for time-to-progression and overall survival in 298 patients with available total metabolic tumor volume data

**Supplementary Table 1. Genotypes and allele frequencies**

| **Polymorphism**  **(rs number)** | **Genotype** | **No. of patients** | **% of**  **patients** | **Allele frequencies** | **Hardy-Weinberg equilibrium, p** |
| --- | --- | --- | --- | --- | --- |
| SLC2A1 HaeIII  45C>T  (rs1385129) | CC  CT  TT | 214  109  19 | 62.6%  31.9%  5.6% | C: 0.785  T: 0.215 | 0.305 |
| SLC2A1 HpyCH4V  -2841A>T  (rs710218) | AA  AT  TT | 210  112  20 | 61.4%  32.7%  5.8% | A: 0.778  T: 0.222 | 0.330 |
| SLC2A1 XbaI  +22999G>T  (rs841853) | GG  GT  TT | 171  143  28 | 50.0%  41.8%  8.2% | G: 0.709  T: 0.291 | 0.804 |
| VEGFA  +936C>T  (rs3025039) | CC  CT  TT | 254  82  6 | 74.3%  24.0%  1.8% | C: 0.863  T: 0.137 | 0.834 |
| HIF1A P582S  1772C>T  (rs11549465) | CC  CT  TT | 283  57  2 | 82.7%  16.7%  0.6% | C: 0.911  T: 0.089 | 0.632 |
| HIF1A A588T  1790G>A  (rs11549467) | GG  GA  AA | 332  10  0 | 97.1%  2.9%  0.0% | G: 0.985  A: 0.015 | 0.784 |
| APEX1 D148E  1349T>G  (rs1130409) | TT  TG  GG | 99  161  82 | 28.9%  47.1%  24.0% | T: 0.525  G: 0.475 | 0.299 |

SLC2A1, solute carrier family 2 member 1; VEGFA, vascular endothelial growth factor A; HIF1A, hypoxia-inducible factor 1A; APEX1, apurinic/apyrimidinic endonuclease 1; p, chi^2^ test

**Supplementary Table 2. APEX1 D148E genotype-dependent association between SLC2A1 genotypes and baseline maximum standardized uptake value**

| **APEX1**  **genotype** | **SLC2A1**  **genotype** | **No.**  **pts.** | **Median SUV_max_**  **(IQR)** | **p** | **Dominant**  **model** | **No.**  **pts.** | **Median SUV_max_**  **(IQR)** | **p** | **Recessive**  **model** | **No.**  **pts.** | **Median SUV_max_**  **(IQR)** | **p** |
| --- | --- | --- | --- | --- | --- | --- | --- | --- | --- | --- | --- | --- |
| **SLC2A1 HaeIII (rs1385129)** | | | | | | | | | | | | |
| TT  (n=99) | CC  CT  TT | 66  30  3 | 21.3 (14.3-32.4)  17.8 (10.7-25.5)  8.3 (n.a.) | 0.038 | CC  CT+TT | 66  33 | 21.3 (14.3-32.4)  16.0 (10.5-25.0) | 0.020 | CC+CT  TT | 96  3 | 10.0 (12.4-31.2)  8.3 (n.a.) | 0.107 |
| TG  (n=160) | CC  CT  TT | 96  54  10 | 20.5 (14.1-26.7)  19.0 (12.0-25.6)  21.5 (11.7-29.9) | 0.651 | CC  CT+TT | 96  64 | 20.5 (14.1-26.7)  19.5 (11.8-26.4) | 0.496 | CC+CT  TT | 150  10 | 20.0 (13.6-26.0)  21.5 (11.7-29.9) | 0.703 |
| GG  (n=82) | CC  CT  TT | 52  24  6 | 22.3 (14.5-30.4)  17.3 (11.6-26.5)  26.6 (16.2-33.3) | 0.183 | CC  CT+TT | 52  30 | 22.3 (14.5-30.4)  18.1 (12.1-30.1) | 0.292 | CC+CT  TT | 76  6 | 21.4 (12.6-30.0)  26.6 (16.2-33.3) | 0.310 |
| **SLC2A1 HpyCH4V (rs710218)** | | | | | | | | | | | | |
| TT  (n=99) | AA  AT  TT | 64  31  4 | 21.3 (14.1-32.5)  17.1 (10.7-25.0)  16.4 (6.4-25.4) | 0.067 | AA  AT+TT | 64  35 | 21.3 (14.1-32.5)  17.1 (10.6-25.0) | 0.024 | AA+AT  TT | 95  4 | 19.9 (12.4-31.2)  16.4 (6.4-25.4) | 0.255 |
| TG  (n=160) | AA  AT  TT | 95  55  10 | 20.4 (14.0-26.9)  19.0 (12.2-25.5)  21.5 (11.7-29.9) | 0.712 | AA  AT+TT | 95  65 | 20.4 (14.0-26.9)  19.9 (12.0-26.2) | 0.565 | AA+AT  TT | 150  10 | 20.0 (13.6-26.0)  21.5 (11.7-29.9) | 0.703 |
| GG  (n=82) | AA  AT  TT | 51  25  6 | 22.2 (14.4-30.4)  17.4 (11.7-27.3)  26.6 (16.2-33.3) | 0.225 | AA  AT+TT | 51  31 | 22.2 (14.4-30.4)  18.7 (12.2-30.1) | 0.349 | AA+AT  TT | 76  6 | 21.4 (12.6-30.0)  26.6 (16.2-33.3) | 0.310 |
| **SLC2A1 XbaI (rs841853)** | | | | | | | | | | | | |
| TT  (n=99) | GG  GT  TT | 54  42  3 | 20.0 (11.0-29.6)  21.4 (13.9-31.9)  12.4 (n.a.) | 0.307 | GG  GT+TT | 54  45 | 20.0 (11.0-29.6)  19.4 (13.6-31.6) | 0.456 | GG+GT  TT | 96  3 | 20.4 (12.3-31.2)  12.4 (n.a.) | 0.240 |
| TG  (n=160) | GG  GT  TT | 75  67  18 | 19.9 (14.5-25.7)  20.4 (12.8-26.6)  19.9 (13.6-28.7) | 0.898 | GG  GT+TT | 75  85 | 19.9 (14.5-25.7)  20.4 (13.1-26.8) | 0.985 | GG+GT  TT | 142  18 | 20.1 (13.6-26.2)  19.9 (13.6-28.7) | 0.658 |
| GG  (n=82) | GG  GT  TT | 41  34  7 | 21.5 (14.9-30.1)  21.0 (11.1-29.9)  28.5 (17.9-33.3) | 0.417 | GG  GT+TT | 41  41 | 21.5 (14.9-30.1)  21.7 (12.0-30.9) | 0.792 | GG+GT  TT | 75  7 | 21.3 (12.8-30.1)  28.5 (17.9-33.3) | 0.249 |

APEX1, apurinic/apyrimidinic endonuclease 1; SLC2A1, solute carrier family 2 member 1; no. pts., number of patients; SUV_max_, maximum standardized uptake value; IQR, interquartile range (25^th^-75^th^ percentile); p, Kruskal-Wallis test; n.a., not applicable

**Supplementary Table 3. Association between genotypes and baseline maximum standardized uptake value**

| **Polymorphism**  **(rs number)** | **Genotype** | **No.**  **pts.** | **Median SUV_max_**  **(IQR)** | **p** | **Dominant**  **model** | **No.**  **pts.** | **Median SUV_max_**  **(IQR)** | **p** | **Recessive**  **model** | **No.**  **pts.** | **Median SUV_max_**  **(IQR)** | **p** |
| --- | --- | --- | --- | --- | --- | --- | --- | --- | --- | --- | --- | --- |
| SLC2A1 HaeIII  (rs1385129) | CC  CT  TT | 214  108  19 | 21.6 (14.4-29.7)  18.6 (11.5-25.5)  22.4 (11.7-30.1) | 0.041 | CC  CT+TT | 214  127 | 21.6 (14.4-29.7)  18.9 (11.5-26.6) | 0.019 | CC+CT  TT | 322  19 | 20.5 (13.3-28.6)  22.4 (11.7-30.1) | 0.878 |
| SLC2A1 HpyCH4V  (rs710218) | AA  AT  TT | 210  111  20 | 21.4 (14.2-29.9)  18.7 (11.5-25.5)  22.8 (12.6-29.9) | 0.055 | AA  AT+TT | 210  131 | 21.4 (14.2-29.9)  19.0 (11.5-26.6) | 0.030 | AA+AT  TT | 321  20 | 20.4 (13.3-28.6)  22.8 (12.6-29.9) | 0.767 |
| SLC2A1 XbaI  (rs841853) | GG  GT  TT | 170  143  28 | 20.1 (13.4-28.8)  20.7 (12.8-29.2)  20.0 (13.3-30.1) | 0.898 | GG  GT+TT | 170  171 | 20.1 (13.4-28.8)  20.7 (13.0-29.2) | 0.898 | GG+GT  TT | 313  28 | 20.5 (13.3-28.8)  20.0 (13.3-30.1) | 0.650 |
| VEGFA  (rs3025039) | CC  CT  TT | 253  82  6 | 20.7 (13.6-29.6)  19.8 (12.1-27.0)  21.2 (14.8-30.5) | 0.258 | CC  CT+TT | 253  88 | 20.7 (13.6-29.6)  19.8 (12.2-27.1) | 0.258 | CC+CT  TT | 335  6 | 20.5 (13.2-28.7)  21.2 (14.8-30.5) | 0.892 |
| HIF1A P582S  (rs11549465) | CC  CT  TT | 282  57  2 | 20.6 (13.4-29.2)  19.9 (11.8-26.1)  16.6 (n.a.) | 0.556 | CC  CT+TT | 282  59 | 20.6 (13.4-29.2)  19.9 (11.3-25.5) | 0.556 | CC+CT  TT | 339  2 | 20.5 (13.3-28.9)  16.6 (n.a.) | 0.488 |
| HIF1A A588T  (rs11549467) | GG  GA  AA | 331  10  0 | 20.5 (13.2-28.9)  16.0 (12.7-29.3)  n.a. | 0.724 | GG  GA+AA | 331  10 | 20.5 (13.2-28.9)  16.0 (12.7-29.3) | 0.724 | GG+GA  AA | 341  0 | 20.5 (13.3-28.8)  n.a. | n.a. |
| APEX1 D148E  (rs1130409) | TT  TG  GG | 99  160  82 | 19.9 (12.2-31.0)  20.1 (13.6-26.5)  21.6 (13.1-30.1) | 0.522 | TT  TG+GG | 99  242 | 19.9 (12.2-31.0)  20.6 (13.6-28.4) | 0.522 | TT+TG  GG | 259  82 | 20.0 (13.3-28.3)  21.6 (13.1-30.1) | 0.378 |

SLC2A1, solute carrier family 2 member 1; VEGFA, vascular endothelial growth factor A; HIF1A, hypoxia-inducible factor 1A; APEX1, apurinic/apyrimidinic endonuclease 1; no. pts., number of patients; SUV_max_, maximum standardized uptake value; IQR, interquartile range (25^th^-75^th^ percentile); p, Kruskal-Wallis test; n.a., not applicable

**Supplementary Table 4. APEX1 D148E genotype-dependent association between SLC2A1 genotypes and Ann Arbor stage IV disease**

| **APEX1**  **genotype** | **SLC2A1**  **genotype** | **No.**  **pts.** | **Ann Arbor**  **Stage I-III vs. IV** | **p** | **Dominant**  **model** | **No.**  **pts.** | **Ann Arbor**  **Stage I-III vs. IV** | **p** | **Recessive**  **model** | **No.**  **pts.** | **Ann Arbor**  **Stage I-III vs. IV** | **p** |
| --- | --- | --- | --- | --- | --- | --- | --- | --- | --- | --- | --- | --- |
| **SLC2A1 HaeIII (rs1385129)** | | | | | | | | | | | | |
| TT  (n=99) | CC  CT  TT | 66  30  3 | 47 vs. 19  13 vs. 17  2 vs. 1 | 0.032 | CC  CT+TT | 66  33 | 47 vs. 19  15 vs. 18 | 0.013 | CC+CT  TT | 96  3 | 60 vs. 36  2 vs. 1 | 0.883 |
| TG  (n=161) | CC  CT  TT | 96  55  10 | 70 vs. 26  29 vs. 26  7 vs. 3 | 0.040 | CC  CT+TT | 96  65 | 70 vs. 26  36 vs. 29 | 0.021 | CC+CT  TT | 151  10 | 99 vs. 52  7 vs. 3 | 0.774 |
| GG  (n=82) | CC  CT  TT | 52  24  6 | 31 vs. 21  16 vs. 8  4 vs. 2 | 0.818 | CC  CT+TT | 52  30 | 31 vs. 21  20 vs. 10 | 0.526 | CC+CT  TT | 76  6 | 47 vs. 29  4 vs. 2 | 0.814 |
| **SLC2A1 HpyCH4V (rs710218)** | | | | | | | | | | | | |
| TT  (n=99) | AA  AT  TT | 64  31  4 | 46 vs. 18  14 vs. 17  2 vs. 2 | 0.036 | AA  AT+TT | 64  35 | 46 vs. 18  16 vs. 19 | 0.010 | AA+AT  TT | 95  4 | 60 vs. 35  2 vs. 2 | 0.594 |
| TG  (n=161) | AA  AT  TT | 95  56  10 | 69 vs. 26  30 vs. 26  7 vs. 3 | 0.056 | AA  AT+TT | 95  66 | 69 vs. 26  37 vs. 29 | 0.029 | AA+AT  TT | 151  10 | 99 vs. 52  7 vs. 3 | 0.774 |
| GG  (n=82) | AA  AT  TT | 51  25  6 | 31 vs. 20  16 vs. 9  4 vs. 2 | 0.938 | AA  AT+TT | 51  31 | 31 vs. 20  20 vs. 11 | 0.735 | AA+AT  TT | 76  6 | 47 vs. 29  4 vs. 2 | 0.814 |
| **SLC2A1 XbaI (rs841853)** | | | | | | | | | | | | |
| TT  (n=99) | GG  GT  TT | 54  42  3 | 32 vs. 22)  28 vs. 14  2 vs. 1 | 0.750 | GG  GT+TT | 54  45 | 32 vs. 22  30 vs. 15 | 0.448 | GG+GT  TT | 96  3 | 60 vs. 36  2 vs. 1 | 0.883 |
| TG  (n=161) | GG  GT  TT | 76  67  18 | 49 vs. 27  46 vs. 21  11 vs. 7 | 0.787 | GG  GT+TT | 76  85 | 49 vs. 27  57 vs. 28 | 0.730 | GG+GT  TT | 143  18 | 95 vs. 48  11 vs. 7 | 0.654 |
| GG  (n=82) | GG  GT  TT | 41  34  7 | 24 vs. 17  23 vs. 11  4 vs. 3 | 0.691 | GG  GT+TT | 41  41 | 24 vs. 17  27 vs. 14 | 0.494 | GG+GT  TT | 75  7 | 47 vs. 28  4 vs. 3 | 0.773 |

APEX1, apurinic/apyrimidinic endonuclease 1; SLC2A1, solute carrier family 2 member 1; no. pts., number of patients; p, chi^2^ test

**Supplementary Table 5. Association between genotypes and Ann Arbor stage IV disease**

| **Polymorphism**  **(rs number)** | **Genotype** | **No.**  **pts.** | **Ann Arbor**  **Stage I-III vs. IV** | **p** | **Dominant**  **model** | **No.**  **pts.** | **Ann Arbor**  **Stage I-III vs. IV** | **p** | **Recessive**  **model** | **No.**  **pts.** | **Ann Arbor**  **Stage I-III vs. IV** | **p** |
| --- | --- | --- | --- | --- | --- | --- | --- | --- | --- | --- | --- | --- |
| SLC2A1 HaeIII  (rs1385129) | CC  CT  TT | 214  109  19 | 148 vs.66  58 vs. 51  13 vs. 6 | 0.017 | CC  CT+TT | 214  128 | 148 vs. 66  71 vs. 57 | 0.011 | CC+CT  TT | 323  19 | 206 vs. 117  13 vs. 6 | 0.682 |
| SLC2A1 HpyCH4V  (rs710218) | AA  AT  TT | 210  112  20 | 146 vs. 64  60 vs. 52  13 vs. 7 | 0.018 | AA  AT+TT | 210  132 | 146 vs. 64  73 vs. 59 | 0.008 | AA+AT  TT | 322  20 | 206 vs. 116  13 vs. 7 | 0.926 |
| SLC2A1 XbaI  (rs841853) | GG  GT  TT | 171  143  28 | 105 vs. 66  97 vs. 46  17 vs. 11 | 0.462 | GG  GT+TT | 171  171 | 105 vs. 66  114 vs. 57 | 0.311 | GG+GT  TT | 314  28 | 202 vs. 112  17 vs. 11 | 0.702 |
| VEGFA  (rs3025039) | CC  CT  TT | 254  82  6 | 165 vs. 89  52 vs. 30  2 vs. 4 | 0.277 | CC  CT+TT | 254  88 | 165 vs. 89  54 vs. 34 | 0.545 | CC+CT  TT | 336  6 | 217 vs. 119  2 vs. 4 | 0.114 |
| HIF1A P582S  (rs11549465) | CC  CT  TT | 283  57  2 | 181 vs. 102  37 vs. 20  1 vs. 1 | 0.909 | CC  CT+TT | 283  59 | 181 vs. 102  38 vs. 21 | 0.948 | CC+CT  TT | 340  2 | 218 vs. 122  1 vs. 1 | 0.678 |
| HIF1A A588T  (rs11549467) | GG  GA  AA | 332  10  0 | 212 vs. 120  7 vs. 3  n.a. | 0.690 | GG  GA+AA | 332  10 | 212 vs. 120  7 vs. 3 | 0.690 | GG+GA  AA | 342  0 | 219 vs. 123  n.a. | n.a. |
| APEX1 D148E  (rs1130409) | TT  TG  GG | 99  161  82 | 62 vs. 37  106 vs. 55  51 vs. 31 | 0.805 | TT  TG+GG | 99  243 | 62 vs. 37  157 vs. 86 | 0.729 | TT+TG  GG | 260  82 | 168 vs. 92  51 vs. 31 | 0.690 |

SLC2A1, solute carrier family 2 member 1; VEGFA, vascular endothelial growth factor A; HIF1A, hypoxia-inducible factor 1A; APEX1, apurinic/apyrimidinic endonuclease 1; no. pts., number of patients; p, chi^2^ test; n.a., not applicable

**Supplementary Table 6. Association between genotypes and baseline total metabolic tumor volume**

| **Polymorphism**  **(rs number)** | **Genotype** | **No.**  **pts.** | **Median TMTV**  **in cm³ (IQR)** | **p** | **Dominant**  **model** | **No.**  **pts.** | **Median TMTV**  **in cm³ (IQR)** | **p** | **Recessive**  **model** | **No.**  **pts.** | **Median TMTV**  **in cm³ (IQR)** | **p** |
| --- | --- | --- | --- | --- | --- | --- | --- | --- | --- | --- | --- | --- |
| SLC2A1 HaeIII  (rs1385129) | CC  CT  TT | 188  95  15 | 146 (35-517)  136 (29-503)  126 (29-409) | 0.771 | CC  CT+TT | 188  110 | 146 (35-517)  127 (29-479) | 0.585 | CC+CT  TT | 283  15 | 145 (34-507)  126 (29-409) | 0.540 |
| SLC2A1 HpyCH4V  (rs710218) | AA  AT  TT | 185  97  16 | 145 (35-514)  136 (30-516)  127 (29-369) | 0.832 | AA  AT+TT | 185  113 | 145 (35-514)  128 (30-482) | 0.630 | AA+AT  TT | 282  16 | 144 (34-510)  127 (29-369) | 0.618 |
| SLC2A1 XbaI  (rs841853) | GG  GT  TT | 151  124  23 | 143 (31-503)  114 (32-449)  230 (44-750) | 0.655 | GG  GT+TT | 151  147 | 143 (31-503)  136 (35-488) | 0.997 | GG+GT  TT | 275  23 | 125 (32-477)  230 (44-750) | 0.379 |
| VEGFA  (rs3025039) | CC  CT  TT | 220  73  5 | 148 (33-563)  73 (30-477)  211 (172-717) | 0.395 | CC  CT+TT | 220  78 | 148 (33-563)  93 (31-477) | 0.532 | CC+CT  TT | 293  5 | 126 (32-497)  211 (172-717) | 0.296 |
| HIF1A P582S  (rs11549465) | CC  CT  TT | 249  47  2 | 145 (32-474)  107 (37-565)  325 (n.a.) | 0.934 | CC  CT+TT | 249  49 | 145 (32-474)  107 (34-582) | 0.792 | CC+CT  TT | 296  2 | 140 (33-490)  325 (n.a.) | 0.042 |
| HIF1A A588T  (rs11549467) | GG  GA  AA | 290  8  0 | 146 (34-510)  16 (7-210) | 0.034 | GG  GA+AA | 290  8 | 146 (34-510)  16 (7-210) | 0.034 | GG+GA  AA | 298  0 | 140 (33-494)  n.a. | n.a. |
| APEX1 D148E  (rs1130409) | TT  TG  GG | 86  143  69 | 239 (31-571)  98 (29-477)  160 (35-517) | 0.448 | TT  TG+GG | 86  212 | 239 (31-571)  112 (34-485) | 0.464 | TT+TG  GG | 229  69 | 128 (31-496)  160 (35-517) | 0.476 |

SLC2A1, solute carrier family 2 member 1; VEGFA, vascular endothelial growth factor A; HIF1A, hypoxia-inducible factor 1A; APEX1, apurinic/apyrimidinic endonuclease 1; no. pts., number of patients; TMTV, total metabolic tumor volume; IQR, interquartile range (25^th^-75^th^ percentile); p, Kruskal-Wallis test; n.a., not applicable

**Supplementary Table 7. Association between genotypes and relative standardized uptake value reduction at interim positron emission tomography**

| **Polymorphism**  **(rs number)** | **Genotype** | **No.**  **pts.** | **Median SUV_max_**  **reduction % (IQR)** | **p** | **Dominant**  **model** | **No.**  **pts.** | **Median SUV_max_**  **reduction % (IQR)** | **p** | **Recessive**  **model** | **No.**  **pts.** | **Median SUV_max_**  **reduction % (IQR)** | **p** |
| --- | --- | --- | --- | --- | --- | --- | --- | --- | --- | --- | --- | --- |
| SLC2A1 HaeIII  (rs1385129) | CC  CT  TT | 214  108  19 | 84.0 (88.8-75.1)  79.9 (87.8-70.6)  82.3 (91.5-76.1) | 0.097 | CC  CT+TT | 214  127 | 84.0 (88.8-75.1)  80.7 (88.3-71.4) | 0.142 | CC+CT  TT | 322  19 | 83.1 (88.4-73.1)  82.3 (91.5-76.1) | 0.298 |
| SLC2A1 HpyCH4V  (rs710218) | AA  AT  TT | 210  111  20 | 84.0 (88.7-75.1)  80.3 (88.0-70.4)  83.0 (91.5-76.8) | 0.126 | AA  AT+TT | 210  131 | 84.0 (88.7-75.1)  80.9 (88.5-71.4) | 0.182 | AA+AT  TT | 321  20 | 83.1 (88.5-73.1)  83.0 (91.5-76.8) | 0.301 |
| SLC2A1 XbaI  (rs841853) | GG  GT  TT | 170  143  28 | 82.4 (88.7-74.7)  83.6 (88.5-73.1)  80.7 (87.8-69.8) | 0.380 | GG  GT+TT | 170  171 | 82.4 (88.7-74.7)  83.4 (88.4-72.5) | 0.815 | GG+GT  TT | 313  28 | 83.4 (88.6-74.1)  80.6 (87.8-69.8) | 0.168 |
| VEGFA  (rs3025039) | CC  CT  TT | 253  82  6 | 83.7 (88.7-74.0)  81.4 (88.0-70.2)  80.9 (84.0-75.9) | 0.380 | CC  CT+TT | 253  88 | 83.7 (88.7-74.0)  81.0 (87.5-70.7) | 0.175 | CC+CT  TT | 335  6 | 83.4 (88.6-73.1)  80.9 (84.0-75.9) | 0.542 |
| HIF1A P582S  (rs11549465) | CC  CT  TT | 282  57  2 | 83.5 (88.8-73.0)  82.0 (87.7-75.7)  70.7 (n.a.) | 0.384 | CC  CT+TT | 282  59 | 83.5 (88.8-73.0)  81.8 (87.7-75.0) | 0.507 | CC+CT  TT | 339  2 | 83.2 (88.6-73.1)  70.7 (n.a.) | 0.190 |
| HIF1A A588T  (rs11549467) | GG  GA  AA | 331  10  0 | 83.1 (88.5-73.1)  84.5 (90.9-69.1)  n.a. | 0.822 | GG  GA+AA | 331  10 | 83.1 (88.5-73.1)  84.5 (90.9-69.1) | 0.822 | GG+GA  AA | 341  0 | 83.1 (88.5-73.1)  n.a. | n.a. |
| APEX1 D148E  (rs1130409) | TT  TG  GG | 99  160  82 | 83.7 (88.5-71.6)  81.8 (88.1-71.1)  84.0 (88.9-77.2) | 0.266 | TT  TG+GG | 99  242 | 83.7 (88.5-71.6)  82.5 (88.6-73.5) | 0.741 | TT+TG  GG | 259  82 | 82.6 (88.4-71.4)  84.0 (88.9-77.2) | 0.171 |

SLC2A1, solute carrier family 2 member 1; VEGFA, vascular endothelial growth factor A; HIF1A, hypoxia-inducible factor 1A; APEX1, apurinic/apyrimidinic endonuclease 1; no. pts., number of patients; SUV_max_, maximum standardized uptake value; IQR, interquartile range (25^th^-75^th^ percentile); p, Kruskal-Wallis test; n.a., not applicable

**Supplementary Table 8. Association between genotypes and interim positron emission tomography response**

| **Polymorphism**  **(rs number)** | **Genotype** | **No.**  **pts.** | **Interim PET**  **Neg. vs. pos.** | **p** | **Dominant**  **model** | **No.**  **pts.** | **Interim PET**  **Neg. vs. pos.** | **p** | **Recessive**  **model** | **No.**  **pts.** | **Interim PET**  **Neg. vs. pos.** | **p** |
| --- | --- | --- | --- | --- | --- | --- | --- | --- | --- | --- | --- | --- |
| SLC2A1 HaeIII  (rs1385129) | CC  CT  TT | 214  109  19 | 194 vs.20  97 vs. 12  17 vs. 2 | 0.891 | CC  CT+TT | 214  128 | 194 vs. 20  114 vs. 14 | 0.634 | CC+CT  TT | 323  19 | 291 vs. 32  17 vs. 2 | 0.930 |
| SLC2A1 HpyCH4V  (rs710218) | AA  AT  TT | 210  112  20 | 191 vs. 19  99 vs. 13  18 vs. 2 | 0.765 | AA  AT+TT | 210  132 | 191 vs. 19  117 vs. 15 | 0.486 | AA+AT  TT | 322  20 | 290 vs. 32  18 vs. 2 | 0.993 |
| SLC2A1 XbaI  (rs841853) | GG  GT  TT | 171  143  28 | 155 vs. 16  130 vs. 13  23 vs. 5 | 0.343 | GG  GT+TT | 171  171 | 155 vs. 16  153 vs. 18 | 0.718 | GG+GT  TT | 314  28 | 285 vs. 29  23 vs. 5 | 0.144 |
| VEGFA  (rs3025039) | CC  CT  TT | 254  82  6 | 231 vs. 23  71 vs. 11  6 vs. 0 | 0.370 | CC  CT+TT | 254  88 | 231 vs. 23  77 vs. 11 | 0.352 | CC+CT  TT | 336  6 | 302 vs. 34  6 vs. 0 | 0.412 |
| HIF1A P582S  (rs11549465) | CC  CT  TT | 283  57  2 | 256 vs. 27  51 vs. 6  1 vs. 1 | 0.161 | CC  CT+TT | 283  59 | 256 vs. 27  52 vs. 7 | 0.587 | CC+CT  TT | 340  2 | 307 vs. 33  1 vs. 1 | 0.058 |
| HIF1A A588T  (rs11549467) | GG  GA  AA | 332  10  0 | 298 vs. 34  10 vs. 0  n.a. | 0.286 | GG  GA+AA | 332  10 | 298 vs. 34  10 vs. 0 | 0.286 | GG+GA  AA | 342  0 | 308 vs. 34  n.a. | n.a. |
| APEX1 D148E  (rs1130409) | TT  TG  GG | 99  161  82 | 90 vs. 9  141 vs. 20  77 vs. 5 | 0.281 | TT  TG+GG | 99  243 | 90 vs. 9  218 vs. 25 | 0.737 | TT+TG  GG | 260  82 | 231 vs. 29  77 vs. 5 | 0.182 |

SLC2A1, solute carrier family 2 member 1; VEGFA, vascular endothelial growth factor A; HIF1A, hypoxia-inducible factor 1A; APEX1, apurinic/apyrimidinic endonuclease 1; PET, positron emission tomography; neg., negative; pos., positive; p, chi^2^ test

**Supplementary Table 9. Cox model for time-to-progression and overall survival in 298 patients with available total metabolic tumor volume data**

|  | **Hazard ratio** | **95% confidence interval** | **p** |
| --- | --- | --- | --- |
| **Time-to-progression** |  |  |  |
| Age >60 years | 1.633 | 1.026-2.598 | 0.037 |
| Ann Arbor stage III or IV | 2.429 | 1.354-4.357 | 0.002 |
| Total metabolic tumor volume >328 cm³ | 2.214 | 1.348-3.637 | 0.002 |
| SLC2A1 XbaI, GT/TT genotypes | 1.572 | 0.991-2.493 | 0.054 |
| APEX1 D148E, GG genotype | 1.641 | 1.009-2.670 | 0.054 |
| **Overall survival** |  |  |  |
| Age >60 years | 2.276 | 1.244-4.164 | 0.006 |
| Lactate dehydrogenase >ULN | 2.326 | 1.216-4.451 | 0.007 |
| B symptoms | 2.911 | 1.619-5.234 | <0.001 |
| SLC2A1 XbaI, GT/TT genotypes | 2.083 | 1.155-3.757 | 0.014 |

SLC2A1, solute carrier family 2 member 1; APEX1, apurinic/apyrimidinic endonuclease 1; ULN, upper limit of normal; p, likelihood ratio test
